# A Database of Drug Repurposing Clinical Trials in Oncology

**DOI:** 10.3389/fphar.2021.790952

**Published:** 2021-11-10

**Authors:** Pan Pantziarka, Liese Vandeborne, Gauthier Bouche

**Affiliations:** ^1^ The Anticancer Fund, Brussels, Belgium; ^2^ The George Pantziarka TP53 Trust, London, United Kingdom

**Keywords:** drug repurposing, oncology, clinical trials, bioinformatics, database

## Introduction

Drug repurposing is a strategy that seeks new medical treatments from existing licensed medications rather than from *de novo* development of new molecules. The rationale for this approach is predicated in part on the perceived advantages of using existing data on safety and toxicity, the ready availability of such medicines and the potential for lowered drug costs (Pantziarka et al., 2014; Bertolini et al., 2015). In particular, the use of non-cancer drugs as new cancer treatments is becoming an increasingly attractive proposition.

While bibliometric data shows an exponential increase in the peer reviewed literature in this area, much of it remains focused on drug candidate identification and pre-clinical studies (Pantziarka et al., 2020). There has been a growth of computational support for candidate identification in recent years, with a focus on algorithms and database support to enhance disease-target-drug analysis (Peyvandipour et al., 2018; Zhu et al., 2018; Tanoli et al., 2021). A notable contribution to the field is the PRISM (profiling relative inhibition simultaneously in mixtures) system from the Broad Institute. This open access resource records the growth inhibitory activity of thousands of compounds against more than 500 cancer cell lines (Corsello et al., 2020). The importance of regulatory approvals, intellectual property rights and other non-clinical factors in repurposing have also been highlighted and discussed, particularly by Verbaanderd et al. (2019) and by Begley et al. (2021).

To date there has not been a comprehensive analysis of clinical trials in repurposing in oncology. The answers to key questions regarding the proportion of clinical trials by drug, by phase, by geographic location or cancer indication remain unclear. This work describes the methodology for the creation of a curated database of drug repurposing clinical trials in oncology. The database, which we have called the ReDO_Trials_DB, is available as an online, open access database with basic search, filtering and download functionality.

Definitions of drug repurposing, sometimes also called drug repositioning, vary widely and can encompass multiple drug development strategies from the further development of previously shelved compounds to the use of licensed cancer drugs in new cancer types to the exploration of non-cancer drugs as new cancer therapeutics (Pushpakom et al., 2019; Pantziarka et al., 2020). In this work we are focused specifically on the use of licensed non-cancer drugs as potential cancer therapeutics. To this end the list of drugs classed as repurposing candidates is derived from the ReDO_DB (https://www.anticancerfund.org/en/redo-db), our previously published drug repurposing database (Pantziarka et al., 2018). To recap, drugs are included in the ReDO_DB if they meet the following criteria:

• Licensed by one or more national/international medicines regulatory agency (e.g., FDA, EMA, MHRA etc)
• Are not licensed for a cancer indication, although drugs used for symptomatic or diagnostic uses in cancer can be included (e.g., anti-emetics) if there is evidence of anticancer activity as well
• Have published evidence of anti-cancer activity, including *in vitro*, *in vivo*, case reports, observational studies or clinical trials

In this paper we present the ReDO_Trials_DB, the methodology employed to generate and maintain it and a first description of the number and characteristics of the included trials.

## Methodology

Clinical trials are eligible for inclusion in the ReDO_Trials_DB if they include one or more of the repurposing candidates from ReDO_DB as an active anticancer agent in an intervention arm. Trials which include the repurposing candidate for supportive care, to address adverse events from other treatments or for other reasons not related to active anticancer uses, are not included in the database. For example, there are many trials of the anti-psychotic drug olanzapine for chemotherapy-induced nausea and vomiting (Chow et al., 2021). While these are repurposing trials in that they are investigating a new use for an existing licensed medication, they are not included in the ReDO_Trials_DB as they are not investigating the anticancer effects of the drug. Similarly, primary cancer prevention trials that use repurposed drugs such as aspirin or metformin are not included, whereas trials assessing these drugs as active cancer treatments are included.

Finally, only active trials are included, these are defined as trials which are recruiting, preparing to recruit or not recruiting anymore but still in progress. Trials which have completed, have been terminated or withdrawn are not included.

The data for clinical trials is sourced from a number of registries *via* ClinicalTrials.gov (NCT), the EU Clinical Trials Register (EUCTR) and the World Health Organisation International Clinical Trials Registry Platform (ICTRP). A semi-automated process is used to generate a single dataset of trials, with duplicate records identified and excluded so that manual assessment of trials can take place.

The list of repurposing candidates is derived from the ReDO_DB. In addition to the international non-proprietary name (INN), the database lists common synonyms for each drug. Each INN and synonym is conjugated with a registry-specific search term to generate automated queries which are used to download and store datasets of clinical trials matching the search terms.

The ClinicalTrials.gov API (https://www.clinicaltrials.gov/ct2/resources/download) is used to download clinical trial records in a tab-separated text format as defined by the API XML schema. This format creates one clinical trial record per row of data in the downloaded file. Search query parameters are used to include interventional trials only, for all trial phases and for all active recruitment statuses for the condition of “cancer.” The data download and processing is performed using custom code in an Excel workbook, which acts as the master file for the database. The process enables an iterative workflow so that repeated queries can be performed to incorporate both new trial registrations and amendments to existing trials to ensure recency and accuracy of data.

Currently there is no API provision for the EUCTR and therefore custom web spider code has been constructed to execute a search for cancer trials for each drug and to download and process the pages for each trial thereby identified. Data is extracted from each downloaded trial HTML page and a dataset constructed by mapping the fields to the same structure as the format derived from NCT.

Finally, the WHO ICTRP includes information from multiple national and international clinical trial registries. API access has been off-line since early in the covid-19 pandemic due to sustained heavy traffic. In consequence the ICTRP now periodically issues weekly updates to their database in the form of comma-separated values text files. The fields in these files have been mapped to the NCT structure and code is used to extract all cancer trials included in them into a single file which acts as the master data file for ICTRP cancer trials in this project. This data file is then used as the search target for queries matching interventional trials to the repurposed drugs. Trials which are included in the ICTRP but derived from the NCT or EUCTR registries are discarded leaving only repurposing trials from other international registries.

**FIGURE 1:**
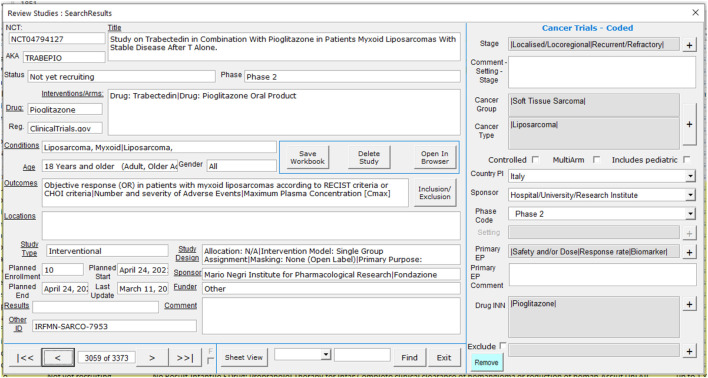
ReDO_Trials_DB coding screen.

Data from all three sources is therefore combined into a single dataset of eligible trials which can then be manually assessed for relevance. Each trial is assessed by one of the authors and coded using a custom interface, as shown in Figure 1. The left section of the screen shows the information downloaded from the trial registry, including links direct to the originating registry should more detailed information be required. The right-hand section is used to record the assessment for the trial. Note that trials which are marked for exclusion are not included in the database, and the reason for exclusion (e.g., drug used for original indication or supportive care) is recorded.

For trials which are deemed to be in-scope, the following information is recorded:

• Stage: Localised/Locoregional, Advanced/Metastatic, Recurrent/Refractory, Any/All Stages, Other (specify)
• Cancer group—a high-level grouping of cancers by histological type: Breast, Urological, Central Nervous System, Gastro-Intestinal, Gynaecological, etc. A trial may include multiple cancer groups
• Cancer type—more detailed selection of cancers within each of the selected cancer groups
• Controlled—Yes/No to indicate if the trial includes a control arm
• Multi-arm—Yes/No to indicate if the trial includes more than one interventional arm
• Includes pediatric—Yes/No to indicate if trial enrolment includes pediatric patients
• Country of principal investigator (PI)
• Type of sponsor—Company, Hospital/University/Research Institute, Collaborative Group, Local/National government, Not available/Missing, Other
• Phase—Phase 1 to Phase 4, Not applicable (N/A), Other
• Setting—For Phase 3 trials only: Primary/Main Curative, Neo-adjuvant/Window, Adjuvant/Maintenance, Perioperative, Palliative or Other
• Primary end-points—One or more of: Safety and/or Dose, Response rate, PFS, OS, DFS/RFS/EFS, Recurrence rate, QoL, Biomarker and Other
• Repurposed drug INN: A delimited list of all the repurposing candidates included in the intervention arms of the trial

**FIGURE 2:**
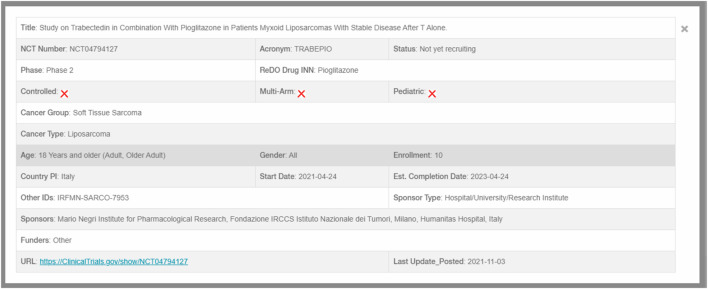
Individual trial record.

Data from all included trials is extracted and uploaded to a SQL database where it is used to populate the online open-access database (https://www.anticancerfund.org/en/redo-trials-db). This allows users to query the database for trials by cancer type, whether trials are controlled and whether pediatric patients are included. Further filtering can be used to include or exclude trials based on drug, trial identifier or trial title. Clicking on an individual trial record displays further details and a link to the original trial registration, as shown in Figure 2.

In addition to the online version, a tab-delimited values text version of the database is also available for download so that the data can be analysed off-line or included in data pipelines such as BioDWH2 (Friedrichs, 2021), CANDO (Schuler et al., 2021) and other bioinformatics platforms.

## Results

As of August 26, 2021 the full dataset consists of 3,734 trials, constructed using 356 drug candidates from the ReDO_DB. Of this dataset of 3,734 trials, 805 have been assessed as being relevant repurposing trials and are therefore included in the ReDO_Trials_DB database. Summary results are shown in Table 1. As of the WHO ICTRP database update of August 16, the total number of active interventional cancer trials of all types is 15,608, which means that repurposing trials are 5.2% of the total. The number of repurposing trials which include pediatric patients is 44 (5.6%). Also recorded in the database is the trial sponsor. These are coded by class of sponsor and the distribution by class is shown in Table 1.

**TABLE 1:** Summary statistics of ReDO_Trials_DB trials.

Trial characteristics	Count	% Of trials
Pediatric patients eligible	44	5.5
Controlled	329	40.9
Multi-arm	93	11.6
Analysis By Phase		
*Phase 1*	146	18.1
*Phase 1/2*	90	11.2
*Phase 2*	353	43.9
*Phase 2/3*	25	3.1
*Phase 3*	82	10.2
*Phase 4*	1	0.1
*Not available/Missing*	31	3.9
*Other*	33	4.1
Analysis By Sponsor Type		
*Company*	48	6.0
*Hospital/University/Research Institute*	701	87.1
*Collaborative Group*	48	6.0
*Local/National government*	5	0.6
*Other*	3	0.4

The distribution of trials by cancer group is shown in Table 2.

**TABLE 2:** Distribution of trials by cancer group.

Cancer group	Count	% Of trials
Gastro-intestinal	204	25.3
Breast	102	12.7
Urological	89	11.1
Central Nervous System	68	8.4
Multiple cancer types	61	7.6
Lung	62	7.7
Gynaecological	45	5.6
Head and Neck	39	4.8
Other hematologic	38	4.7
Leukaemia	37	4.6
Skin	26	3.2
Lymphoma	26	3.2
Bone Sarcoma	18	2.2
Soft Tissue Sarcoma	18	2.2
Other	22	2.7

Of the 356 repurposing drugs, there are clinical trials involving 164 (46%) of them. Out of the 122 ReDO drugs included in the WHO Essential Medicines List, 71 (58%) have active clinical trials listed in the ReDO trials database. In terms of the patent status, the ReDO database shows 287 drugs (80.6%) are off-patent, which is similar to the ReDO trials database with 134 of the 164 (81.7%) trials investigating ReDO drugs being off-patent.

The most popular drugs in terms of number of trials are shown in Table 3. Note that some trials may include more than one repurposing candidate.

**TABLE 3:** Most popular drugs by trial.

Drug	Number	% Of trials
Metformin	128	15.9
Celecoxib	49	6.1
Hydroxychloroquine	45	5.6
Acetylsalicylic Acid	38	4.7
Ascorbic acid	34	4.2
Sirolimus	28	3.5
Valproic Acid	29	3.6
Zoledronic Acid	23	2.9
Propofol	24	3.0
Cholecalciferol	22	2.7
Propranolol	18	2.2
Clarithromycin	19	2.4
Tocilizumab	18	2.2
Simvastatin	18	2.2
Atorvastatin	17	2.1

**FIGURE 3:**
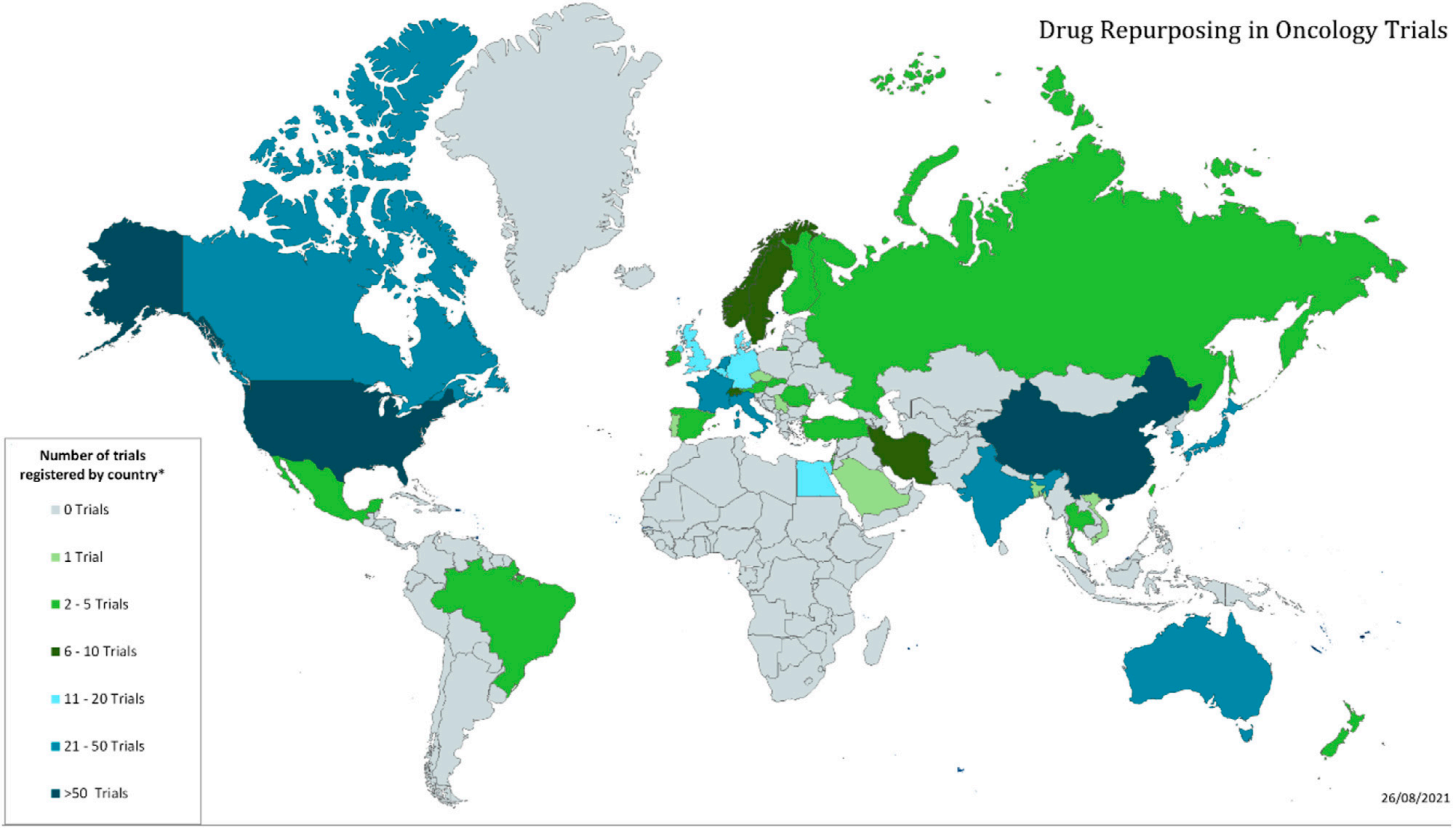
Global distribution of repurposing trials in oncology.

While we do not collect information on the trial locations for each trial, we do record the country of the PI for each trial. In all there are trials originating in 41 different countries. The number of trials in the top 15 countries are shown in Table 4, and the global distribution is shown in Figure 3.

**TABLE 4:** Trials by country.

Country	Count	% Of trials
United States	317	39.4
China	92	11.4
India	45	5.6
Netherlands	38	4.7
Italy	37	4.6
Japan	28	3.5
Canada	24	3.0
Australia	22	2.7
France	21	2.6
Korea, Republic of	21	2.6
United Kingdom	18	2.2
Denmark	17	2.1
Egypt	14	1.7
Belgium	12	1.5
Germany	12	1.5

The planned accrual of patients to all the trials in the database is 157,342 with an average of 206 patients per included trial.

## Discussion

The field of drug repurposing in oncology encompasses a wide range of scientific and clinical research but to date there has not been a dedicated source of information on clinical trials in repurposing. The ReDO_Trials_DB is the first database of repurposing trials focused solely on interventional trials of non-cancer drugs as new cancer treatments.

The current release of the database includes 805 active trials, which represents an estimated 5.2% of all active oncology trials. It is often asserted that an advantage of drug repurposing is that early phase trials may be avoided as existing data on safety and tolerability obviates the need for them. The data here shows that 18.1% of repurposing trials are at phase 1 and a further 11.2% are classified as phase 1/2. Another putative advantage of repurposing is the availability and low-cost of many repurposing candidates, shown here in the high proportion (81.7%) of drugs included in the clinical trials having off-patent status. However, it is also interesting to note that some generic drug candidates, particularly metformin, are included in many trials, suggesting a degree of duplication is likely. The inclusion of pediatric patients in repurposing trials is low, with only 44 (5.5%) of trials open to pediatric patients.

In addition to building the database, a process has been developed to maintain the currency of the data. The iterative process described previously enables updates to existing trial records to be incorporated into the database and new trials added. The process also caters for withdrawal of trials—for trials completing or being terminated early. Such trials will be flagged and tracked so that we can maintain a longitudinal view of trial activity. A research question we wish to explore in the future is an assessment of the completion and reporting rates of repurposing trials.

The database has a number of strengths and weaknesses. A key strength is the manual coding process which enables expert reviewers to assess a trial for inclusion and to code key data regarding interventions, end-points etc. This semi-automated process can make up for data quality issues regarding the data from the registries—particularly with inconsistent trial naming, identification of multiple registrations, and inconsistent use of trial phase identifiers.

One weakness is that data quality issues can remain in the data even after manual review. Duplicate records may be identified and only one selected for inclusion, but there are cases where there is different information in the duplicate records—due to the data being updated to the registries at different time points for example. Another issue is that the choice of repurposing candidates is via the ReDO_DB and there may be relevant repurposing trials testing drugs which are not yet included in that database. In cases where such trials do come to attention, the drug will be added to the ReDO_DB at the next update and then included in the ReDO_Trials_DB at a subsequent release. Finally, the focus on active trials means that we cannot comment on any long-term trends in terms of increasing or decreasing numbers of trials, changes in trial characteristics over time and so on. However, as previously mentioned, our prospective tracking of trial completion will, in time, allow the database to be used to analyse such trends.

In addition to maintaining the currency of the data, the development of the database will continue so that additional data fields and types of analyses may be included in the future. In particular, it is hoped that the data from the database can be incorporated into bioinformatics pipelines for the identification of new candidates for specific cancer types.

## Data Availability

The datasets presented in this study can be found in online repositories. The names of the repository/repositories and accession number(s) can be found below: https://acfdata.coworks.be/ReDO_Trials_DB.txt

## Abbreviations

API, Application Programming Interface; DFS, Disease Free Survival; EFS, Event Free Survival; EMA, European Medicines Agency; FDA, Food and Drug Administration; MHRA, Medicines and Healthcare products Regulatory Agency; OS, Overall Survival; PFS, Progression Free Survival; QoL, Quality of Life; RFS, Recurrence Free Survival; SQL, Structured Query Language.
